# Closing the Solidarity Gap Requires Closing the Accountability Gap for Patient-Facing AI

**DOI:** 10.2196/108685

**Published:** 2026-09-08

**Authors:** Benjamin J Galatzan, Katherine M Dudding, Elizabeth A Johnson

**Affiliations:** 1 Capstone College of Nursing, University of Alabama, 650 University Blvd E, Tuscaloosa, AL, 35401, United States, 1 719 502 6600; 2 College of Nursing, Montana State University, Bozeman, MT, United States

**Keywords:** chatbots, artifical intelligence, AI, generative AI, policy, health care provider

## Abstract

This commentary extends recent discussion of the solidarity gap associated with patient-facing AI by examining gaps in governance and risk allocation, health care professionals’ responsibilities in practice, and opportunities for professional stewardship and advocacy. We argue that equitable implementation requires shared accountability and meaningful health care professional participation in the design, evaluation, reimbursement, governance, and oversight of patient-facing AI before ambiguity results in patient harm.

## Introduction

Barnhart et al [1] described the potential for what is expressed as a solidarity gap created by patient-facing AI tools such as ChatGPT Health. On the surface, these tools appear to address gaps in access to health information and support. However, they can amplify existing inequities in care resources and quality, creating potential for unsafe triage, automation bias, lack of nuance in communication, and insufficient human oversight [1]. The purpose of this commentary is to expand the discussion of the solidarity gap by addressing 3 areas not fully examined in the original article: the regulatory and legislative context surrounding patient-facing AI, the responsibilities of health care providers (HCPs) in practice, and opportunities for proactive engagement to reduce the solidarity gap.

## Solidarity in Governance and Risk Allocation

The solidarity gap, that is, the disconnect between digital health’s promised shared benefits and clinical realities, has growing legal, regulatory, and professional consequences amid the United States’ record care costs and widespread service closures. HCPs remain bound by an overarching duty to care that requires independent clinical judgment, regardless of whether AI contributes to decision-making. Embedded within this duty are jurisprudential responsibilities related to appropriate delegation, assessment of fitness for purpose, and accountability for patient outcomes. However, unlike several European regulatory approaches that more explicitly distribute responsibility across developers, deployers, and health care organizations, the United States continues to rely largely on HCPs’ licensure as the principal locus of accountability. As AI systems become increasingly embedded within clinical workflows, clinicians may have limited practical ability to opt out of AI-supported processes while continuing to bear primary legal and professional responsibility for downstream decisions. This creates an asymmetry in which adoption is increasingly organizational, whereas liability remains largely individual. Current oversight by state medical and nursing boards provides limited guidance regarding evaluation of AI tools outside their intended use, assessment of source validity, documentation expectations, or recommended responses when AI-generated recommendations conflict with clinical judgment. Consequently, HCPs must often determine fitness for purpose without standardized competencies, governance structures, or legal protections that proportionately distribute risk. The resulting imbalance illustrates an important manifestation of the solidarity gap: AI implementation has progressed more rapidly than the development of corresponding frameworks for shared accountability. In an independent assessment of ChatGPT Health, Ramaswamy et al [2] found that overall performance was strong for many routine clinical scenarios; however, safety failures emerged at clinically consequential extremes. Notably, the model undertriaged 51.6% of true emergencies while overtriaging 64.8% of nonurgent presentations. The authors identified inconsistent recognition of suicidal ideation requiring crisis intervention, suggesting diminished reliability when clinical urgency depended on disease progression rather than classic presentation. Recommendations to seek evaluation by an HCP are particularly consequential where shortages of mental health and emergency services limit secondary clinical review, delay needed care, and erode trust when recommended resources are inaccessible. These findings add to growing evidence that governance mechanisms have not kept pace with the shift from AI as a largely self-regulating industry into one integrated within the heavily regulated health care system.

## Solidarity in Practice Economics and Workforce Infrastructure

Solidarity means shared accountability for the business decisions and resources that sustain care delivery among all who shape health care, from individual HCPs to vendors and policymakers. Barnhart et al [1] argue that patient-facing AI should complement human care and be embedded within accountable systems. What remains less developed is the responsibility HCPs have for how tools affect care within their own practice communities. HCPs should function as active co-designers and leaders in translating practical knowledge within technology implementation rather than as end users expected to adapt after deployment [3]. This involvement is particularly important because clinicians may remain responsible for outcomes influenced by AI systems they did not design, may not fully understand, and may lack authority to override [4]. Emerging reimbursement proposals further underscore the need for HCP co-design and leadership in AI-generated clinical analysis payment models. Patient-facing AI may further reshape health care delivery by reducing billable encounters while scaling beyond the constraints related to specific facilities, geographic areas, and clinical privileges that govern clinical care. Broader Medicare reforms seek to reward longitudinal, coordinated, and preventive care; however, they do not yet clearly define how patient-facing AI should be incorporated into quality measurement, attribution models, or shared accountability frameworks.

## Solidarity in Professional Stewardship and Advocacy

Advocacy can help close the solidarity gap by demanding accountability spanning from individuals to the enterprises that shape professional governance and the interpretation of care outcomes. Advocacy extends professional responsibility beyond practice, requiring HCPs to engage in policy discussions rather than passively await new governance structures. Participation in institutional government affairs offices, professional societies, specialty organizations, and state and federal advocacy initiatives provides opportunities to influence legislation, reimbursement policy, and regulatory guidance. These established channels already shape payment policy, licensure, and quality standards and therefore represent natural venues for advancing AI governance. Existing federal initiatives likewise provide opportunities to address structural inequities that predate AI but will influence its implementation. The proposed Creating Opportunities Now for Necessary and Effective Care Technologies (CONNECT) for Health Act would extend access to telehealth and other digitally enabled care beyond temporary emergency authorities [5]. The Centers for Medicare and Medicaid Services Rural Health Transformation Program offers HCPs an opportunity to help shape statewide care delivery reform [6]. Education for practitioners in the ability to interpret legislation impacting or widening acceptable risk tolerance as well as model cards or assurance standards will be critical in their ability to explain rationales for AI tool use or discrepancies.

## Conclusion

Ultimately, solidarity is the collective pursuit of clarity before ambiguity becomes harm. Patient-facing AI presents an opportunity to expand access to care, but technological innovation alone will not achieve equitable, trustworthy health care. As Barnhardt et al [1] argue, solidarity requires shared responsibility. We extend this principle by proposing that solidarity must also encompass shared governance, accountability, and meaningful participation by HCPs in the design, implementation, evaluation, reimbursement, and oversight of patient-facing AI.

## Abbreviations

HCP: health care professional

## References

[1] Barnhart AJ, Comerci G, Prainsack B, Braun M (2026). Solidarity or segregation? ChatGPT Health and US health care disparities. J Med Internet Res.

[2] Ramaswamy A, Tyagi A, Hugo H (2026). ChatGPT Health performance in a structured test of triage recommendations. Nat Med.

[3] Johnson EA, Galatzan BJ (2025). A critical juncture: reimagining nursing professional identity and regulation in the ethical integration of innovation and technology in healthcare. J Nurs Regul.

[4] Galatzan BJ, Johnson EA, Turchioe MR, Baker C, Wieben A (2025). Regulating at the AI frontier: the collision of policy, regulation, and nursing practice. J Nurs Regul.

[5] (2025). H.r.4206 - CONNECT for Health Act of 2025. US Congress.

[6] (2026). Rural Health Transformation (RHT) program. Centers for Medicare and Medicaid Services.

